# Risk factors for cardiovascular disease in patients with metabolic-associated fatty liver disease: a machine learning approach

**DOI:** 10.1186/s12933-022-01672-9

**Published:** 2022-11-12

**Authors:** Karolina Drożdż, Katarzyna Nabrdalik, Hanna Kwiendacz, Mirela Hendel, Anna Olejarz, Andrzej Tomasik, Wojciech Bartman, Jakub Nalepa, Janusz Gumprecht, Gregory Y. H. Lip

**Affiliations:** 1 Department of Internal Medicine, Diabetology and Nephrology, Faculty of Medical, Sciences in Zabrze, Medical University of Silesia, 3 Maja 13-15, 41-800 Zabrze, Katowice, Poland; 2 Liverpool Centre for Cardiovascular Science at University of Liverpool, Liverpool John Moores University and Liverpool Heart & Chest Hospital, Liverpool, UK; 3 Students’ Scientific Association By the Department of Internal Medicine, Diabetology and Nephrology in Zabrze, Faculty of Medical Sciences in Zabrze, Medical University of Silesia, Katowice, Poland; 4 Second Department of Cardiology, Faculty of Medical Sciences in Zabrze, Medical University of Silesia, Katowice, Poland; 5 Department of Neurology, Faculty of Medical Sciences in Zabrze, Medical University of Silesia, Katowice, Poland; 6 Department of Algorithmics and Software, Silesian University of Technology, Gliwice, Poland; 7 Department of Clinical Medicine, Aalborg University, Aalborg, Denmark

**Keywords:** Cardiovascular disease, Metabolic-associated fatty liver disease, Machine learning

## Abstract

**Background:**

Nonalcoholic fatty liver disease is associated with an increased cardiovascular disease (CVD) risk, although the exact mechanism(s) are less clear. Moreover, the relationship between newly redefined metabolic-associated fatty liver disease (MAFLD) and CVD risk has been poorly investigated. Data-driven machine learning (ML) techniques may be beneficial in discovering the most important risk factors for CVD in patients with MAFLD.

**Methods:**

In this observational study, the patients with MAFLD underwent subclinical atherosclerosis assessment and blood biochemical analysis. Patients were split into two groups based on the presence of CVD (defined as at least one of the following: coronary artery disease; myocardial infarction; coronary bypass grafting; stroke; carotid stenosis; lower extremities artery stenosis).

The ML techniques were utilized to construct a model which could identify individuals with the highest risk of CVD. We exploited the multiple logistic regression classifier operating on the most discriminative patient’s parameters selected by univariate feature ranking or extracted using principal component analysis (PCA). Receiver operating characteristic (ROC) curves and area under the ROC curve (AUC) were calculated for the investigated classifiers, and the optimal cut-point values were extracted from the ROC curves using the Youden index, the closest to (0, 1) criteria and the Index of Union methods.

**Results:**

In 191 patients with MAFLD (mean age: 58, SD: 12 years; 46% female), there were 47 (25%) patients who had the history of CVD. The most important clinical variables included hypercholesterolemia, the plaque scores, and duration of diabetes. The five, ten and fifteen most discriminative parameters extracted using univariate feature ranking and utilized to fit the ML models resulted in AUC of 0.84 (95% confidence interval [CI]: 0.77–0.90, *p* < 0.0001), 0.86 (95% CI 0.80–0.91, *p* < 0.0001) and 0.87 (95% CI 0.82–0.92, *p* < 0.0001), whereas the classifier fitted over 10 principal components extracted using PCA followed by the parallel analysis obtained AUC of 0.86 (95% CI 0.81–0.91, *p* < 0.0001). The best model operating on 5 most discriminative features correctly identified 114/144 (79.17%) low-risk and 40/47 (85.11%) high-risk patients.

**Conclusion:**

A ML approach demonstrated high performance in identifying MAFLD patients with prevalent CVD based on the easy-to-obtain patient parameters.

## Background

According to the statistics from the Global Burden of Disease study 2017, a significant number of all deaths globally (over 70%) is caused by noncommunicable diseases, and CVD accounts for more than 43% [1]. The global prevalence of the non-alcoholic fatty liver disease (NAFLD) affects about 1 billion people worldwide [2] and patients with type 2 diabetes (T2DM) constitute the majority of cases [3]. NAFLD is the most rapidly increasing cause of liver-related mortality across the globe [3]. However, it is not the liver itself but CVD that is the leading cause of death in patients with NAFLD [4].

For the last 40 years, NAFLD was characterized as an excessive hepatic lipid accumulation associated with metabolic abnormalities in the absence of significant alcohol consumption and other known causes of liver disease [5, 6]. Because NAFLD has been increasingly associated with glucose and lipid metabolic abnormalities as well as cardiovascular risk that is why modification in the definition was proposed in early 2000s [7, 8]. Thus, the nomenclature changed from NAFLD to the metabolic-associated fatty liver disease (MAFLD) in 2020 [9] and there is increasing evidence proving its importance in multidisciplinary care [10, 11]. Since the diagnosis of MAFLD is relatively new and it differs from NAFLD as it requires the presence of the metabolic risk factors and does not require the exclusion of alcohol intake or the presence of other liver diseases, the clinical course of the disease in patients with MAFLD might be different from NAFLD.

It is important to note that both European and American guidelines recommend to screen for CVD in people with NAFLD [5, 12] and it was demonstrated recently that better prediction of the progression of atherosclerotic cardiovascular risk is obtained when the MAFLD (not NAFLD) definition is used [13, 14] Therefore, it is of paramount clinical importance to determine the factors associated with CVD in people with MAFLD because the CVD could be prevented if an efficient tool for early detection of the individuals at the highest risk was available.

Predicting CVD events within the next 10 years with the use of the traditional risk factors is commonly applied [15]. However, numerous studies have shown that the currently adopted 10-year risk calculators, including the 2013 American College of Cardiology/American Heart Association (ACC/AHA) Pooled Cohort Equations Risk Calculator [15], often overestimate the CVD events and in some cases, underestimate the risk [16–19], causing unnecessary prescriptions of drugs. Moreover, chronic CVD generate high socioeconomic cost, and hence it is a major public health task to find and manage people with risk factors before the onset of CVD, facilitating effective prevention strategies [20].

Machine learning (ML) is one method of artificial intelligence in which computers utilize statistical approaches to effectively learn from data without being explicitly programmed to tackle a specific task. A variety of ML techniques have been increasingly applied in the medical field, including for the prediction of ventilator-associated pneumonia in critical care patients [21], prolonged operative time in elective total shoulder arthroplasty [22], cancer-associated deep vein thrombosis [23], or the stroke risk in non-anticoagulated patients with and without atrial fibrillation [24]. In a recent study, Kakadiaris et al. showed that their ML Risk Calculator outperformed the ACC/AHA Risk Calculator, and proposed less drug therapies while missing fewer CVD events [25].

We applied ML to determine the MAFLD patients who are at high CVD risk, and to better define the underlying risk factors in a data-driven manner. To the best of our knowledge, there have been no studies performed to date that analyzed the associations between MAFLD and CVD risk using ML methods.

## Methods

This single center, observational, study was performed in a cohort of MAFLD patients. Patients who had been previously diagnosed with fatty liver disease based on the liver ultrasonography were invited to participate in the study through the advertisement placed in the University Hospital in Zabrze and in the outpatient diabetology clinics and family doctor clinics in the Upper Silesia region in Poland. The eligibility criteria were simple, ie. patients at age  ≥ 18 years who fulfilled diagnostic criteria of MAFLD. The only exclusion criteria was the lack of written informed consent for the participation in the study. We recorded demographic and clinical data (Table 1) and divided the patients in relation to the presence or absence of CVD. The presence of CVD was defined as one or more of the following: angiography-confirmed coronary artery disease; myocardial infarction; coronary bypass grafting (CABG); stroke; carotid stenosis of at least 50% in diameter; and/or angiography-confirmed, clinically-significant, lower extremities artery stenosis (peripheral artery disease). Every patient signed an informed consent agreement for participation in the study. The study protocol obtained the approval of the Ethics Committee by the Medical University of Silesia (KNW/0022/KB1/38/I/17).

**Table 1:** Patient characteristics

Parameter	Patients without CVD (n = 144)	Patients with CVD (n = 47)	p-value
Biochemical parameters			
ALT [U/l]	47.92 ± 38.60 (32.75)	32.15 ± 15.44 (31.00)	0.0330
AST [U/l]	38.40 ± 33.93 (28.10)	28.83 ± 11.35 (25.80)	0.1342
eGFR [ml/min/1.73m^2^]	88.98 ± 18.52 (90.86)	76.31 ± 17.92 (78.08)	< 0.0001
HbA1c [%]	6.70 ± 1.73 (6.10)	7.30 ± 1.76 (6.85)	0.0025
HDL-C[mmol/l]	1.33 ± 0.57 (1.23)	1.25 ± 0.39 (1.19)	0.4144
HOMA-IR	7.58 ± 6.79 (5.80)	6.94 ± 5.59 (5.89)	0.7293
TG [mmol/l]	2.13 ± 1.95 (1.66)	2.90 ± 3.05 (1.95)	0.1220
Total cholesterol [mmol/l]	5.13 ± 1.57 (4.80)	4.92 ± 1.98 (4.60)	0.0839
Demographical parameters			
Age [years]	56.03 ± 12.15 (57.00)	65.68 ± 6.37 (66.00)	< 0.0001
Gender (% of male)	52.78	57.45	0.5771
Clinical parameters			
ACEi or ARB (% of yes)	49.31	65.96	0.0469
Active smoker (% of yes)	14.58	17.02	0.6859
Betablocker (% of yes)	36.81	65.96	0.0005
BMI [kg/m^2^]	33.74 ± 4.83 (33.39)	32.50 ± 4.83 (31.28)	0.0415
Diastolic BP [mmHg]	81.83 ± 9.97 (80.00)	78.91 ± 9.14 (80.00)	0.0314
T2DM (% of yes)	58.33	85.11	0.0008
Duration of T2DM [years]	3.76 ± 5.60 (0.50)	8.90 ± 7.69 (10.00)	< 0.0001
Hypercholesterolemia (% of yes)	27.08	70.21	< 0.0001
Hypertriglyceridemia (% of yes)	7.64	10.64	0.5193
Heart rate [beats/min]	78.15 ± 15.84 (75.00)	74.04 ± 8.96 (72.00)	0.1940
Hypertension (% of yes)	70.14	93.62	0.0011
Obesity (% of yes)	78.47	65.96	0.0837
Overweight (% of yes)	18.75	31.91	0.0585
Systolic BP [mmHg]	132.70 ± 13.74 (130.00)	133.60 ± 10.67 (132.00)	0.5461
WHR	0.97 ± 0.10 (0.97)	0.99 ± 0.09 (0.99)	0.3318
Carotid ultrasound parameters			
Diameter of CCA L	7.37 ± 0.85 (7.30)	7.61 ± 1.40 (7.70)	0.0192
Diameter of CCA R	7.71 ± 0.95 (7.70)	7.92 ± 0.95 (7.90)	0.2446
IMT max CCA L	0.83 ± 0.18 (0.83)	0.85 ± 0.15 (0.84)	0.4248
IMT max CCA R	0.82 ± 0.18 (0.80)	0.85 ± 0.17 (0.81)	0.2159
Median IMT CCA R	0.68 ± 0.16 (0.66)	0.68 ± 0.15 (0.68)	0.8730
Median IMT CCA L	0.68 ± 0.16 (0.68)	0.71 ± 0.13 (0.69)	0.1660
Plaque area L	0.04 ± 0.07 (0.00)	0.16 ± 0.29 (0.08)	< 0.0001
Plaque area R	0.06 ± 0.23 (0.00)	0.15 ± 0.22 (0.08)	< 0.0001
Plaque score L	0.76 ± 1.33 (0.00)	2.24 ± 1.92 (2.00)	< 0.0001
Plaque score R	0.89 ± 1.47 (0.00)	2.23 ± 1.86 (1.90)	< 0.0001
Arterial stiffness-related parameters			
cfPWV	9.08 ± 2.03 (8.85)	10.30 ± 2.42 (10.10)	< 0.0001
crPWV	9.80 ± 1.55 (9.84)	9.73 ± 1.46 (9.60)	0.4637
Liver elastography parameters			
Liver steatosis CAP [dB/m]	316.30 ± 51.50 (323.00)	315.20 ± 43.76 (311.00)	0.4740
Steatosis stage (S0-S3)	2.36 ± 0.87 (3.00)	2.28 ± 0.77 (2.00)	0.2729

For each parameter (if applicable), we report its mean ± standard deviation, together with the median (in parentheses)

*CVD* cardiovascular disease, *ALT* alanine aminotransferase, *AST* aspartate aminotransferase, *BP* blood pressure, *eGFR* estimated glomerular filtration rate, *HbA1c* hemoglobin A1c, *HOMA-IR* homeostasis model assessment of insulin resistance, *HDL-C* high density lipoprotein cholesterol, *TG* triglycerides, *ACEi* angiotensin converting enzyme inhibitor, *ARB* angiotensin receptor blocker, *MALFD* metabolic-associated fatty liver disease, *BMI* body mass index, , *CCA L* left common carotid artery, *CCA R* right common carotid artery, *IMT* intima -media thickness, *cfPWV* carotid- femoral pulse wave velocity, *crPWV* carotid-radial pulse wave velocity, *CAP* controlled attenuation parameter, *T2DM* type 2 diabetes mellitus.

The p-values were obtained using the Mann–Whitney test or the Chi-square test, depending on the data characteristics

### Anthropometric, demographic and clinical parameters

Anthropometric parameters, including height (meters) and weight (kilograms), as well as waist and hip circumference were measured (meters) by standard methods, and the body mass index (BMI) was calculated as weight/height^2^ (kg/m^2^). Additionally, the waist-to-hip ratio (WHR) was obtained. The obesity was diagnosed when BMI ≥ 30 whereas the overweight was diagnosed when BMI ≥ 25 but < 30. The patients were considered to have T2DM based on a known history of this disease. Blood pressure was measured three times after 5 min of rest in a sitting position, at least 2 min apart, and the mean blood pressure of the three measurements was calculated. Arterial hypertension was defined as a systolic blood pressure ≥ 140 mmHg and/or a diastolic blood pressure ≥ 90 mmHg or previous treatment with antihypertensive medications. Hypercholesterolemia was recognized when a patient had this diagnosis present in the documented medical history and/or there was newly recognized plasma high density lipoprotein cholesterol (HDL-C) < 1.0 mmol/l for men and < 1.3 mmol/l for women and/or patient was on statin therapy. Hypertriglyceridemia was recognized when a patient had this diagnosis present in the documented medical history and/or there was newly recognized plasma triglyceride ≥ 1.7 mmol/l and/or patient was on fibrate therapy. The history of all concomitant diseases was obtained from the patient and confirmed on the basis of documented medical data. No self-reported diseases without medically confirmed diagnosis were recorded.

### MAFLD diagnostic criteria

The patients were diagnosed with MAFLD [26] if there was an evidence of steatosis acquired by the hepatic ultrasonography and presence of one of the following criteria: T2DM, overweight or obesity defined as BMI greater than or equal to 25 kg/m^2^ or at least two metabolic risk abnormalities, i.e., waist circumference ≥ 102 cm in men and ≥ 88 cm in women, blood pressure ≥ 130/85 mmHg or specific drug treatment, prediabetes, plasma triglycerides ≥ 1.7 mmol/l or specific drug treatment, plasma HDL-C < 1.0 mmol/l for men and < 1.3 mmol/l for women or specific drug treatment,  Homeostatic Model Assessment of Insulin Resistance (HOMA- IR) ≥ 2.5, serum C-reactive protein level > 2 mg/l.

## Liver elastography with Fibroscan

Liver elastography was performed with the use of the Fibroscan 502TOUCH F611100049 device exploiting the XL 8 80,685 probe (2.5 Hz). The liver was tested after 6 h of fasting, and the test lasted from 5 to 10 min. In the patient lying in the dorsal position with the right arm extended, a gel-coated ultrasound probe was applied to the skin in the intercostal space above the right lobe of the liver. The time motion ultrasound image allows the operator to locate a fragment of the liver at least 6 cm thick and devoid of large vascular structures or ribs. The median and interquartile range (IQR) values of at least 10 successful liver stiffness measurements (LSM) and the controlled attenuation parameter (CAP), adequately defining fibrosis and steatosis, respectively, were calculated by the device. The results included the median and IQR of the CAP values (dB/m) and the median, IQR, IQR/median LSM (kPa), the success rate (i.e., the number of successful measurements/total number of attempts), the determination of the degree of steatosis (S0-S3) and liver fibrosis (F0-F4). The LSM classified as reliable are characterized by having all three of the following: ≥ 10 passed measurements, ≥ 60% success rate, and IQR/ median < 0.30.

### Carotid ultrasound measurement

The ultrasound examination of the carotid arteries was performed using a high-resolution Doppler ultrasound with double imaging and color coding of the flow (Color Doppler Duplex, CDD) exploiting the Esaote MyLab60 ultrasound equipment by the same certified neurologist. The examinations were done in the supine position, without any additional preparation, with a linear ultrasound head emitting an ultrasonic wave with a variable frequency of 4 MHz to 15 MHz. In the 2D presentation, the common carotid artery (CCA), the separation (bifurcation) of the common carotid artery into the internal carotid artery and the external and internal carotid artery (ICA), alongside the external carotid artery (ECA) were determined. Measurement of the intima-media complex (KIM) thickness and the assessment of atherosclerotic lesions in the carotid arteries were performed.

### Arterial stiffness assessment

For these measurements, piezoelectric mechanical transducers in the cervical, femoral and radial areas were used (Complior, Alam Medical, France). The velocity of the carotid-femoral pulse wave (cfPWV) was used to assess the stiffness of the central artery and the velocity of the carotid-radial pulse wave (crPWV) was used to assess the stiffness of the peripheral arteries. With the Seca mod. 207 height meter, the right carotid-femoral and carotid-radial distances were measured. Blood pressure was measured in the supine position after at least 5 min of rest with the Microlife BP A1 sphygmomanometer immediately prior to the PWV assessment, and the mean of the three measurements on both arms was calculated and recorded. Derivative variables such as the central blood pressure, central pulse pressure and the gain index were analyzed, calculated by the integrated software on the basis of the carotid pulse waveform.

### Biochemical methods

Hemoglobin A1c (HbA1c) was determined using a high-performance liquid chromatography (HPLC) method, and the results were expressed in the National Glycohemoglobin Standardization Program/Diabetes Control and Complications trial units [27]. Cholesterol and triglycerides were measured using the enzymatic methods, with the HDL-C measured after precipitation of the very low-density lipoprotein cholesterol (VLDL-C). The concentration of the low density lipoprotein cholesterol (LDL-C) was calculated using the Friedewald formula [28]. Serum creatinine was measured by means of the Jaffe’s method. The estimated glomerular filtration rate (eGFR) per 1.73 m^2^ was calculated according to the Chronic Kidney Disease Epidemiology Collaboration (CKD-EPI) formula [29]. Blood cell morphology to obtain the platelet count (PLT) was determined using the fluorescent flow cytometry with the Sysmex XN-1000 (Sysmex) hematology analyzer [30]. Serum C reactive protein concentration were measured by a latex particle-enhanced turbidimetric immunoassay [31]. Alanine and aspartate aminotransferase activities in serum were assayed by the kinetic method according to the IFCC reference procedure. Analyzes of serum C reactive protein, alanine and aspartate aminotransferase were carried out on the Cobas 6000 analyzer, c 501 module (Roche). Fasting glucose was assessed using the enzymatic method with the Cobas 6000 hexokinase analyzer, c501 module (Roche). Insulin concentration was measured by electrochemiluminescence using the Cobas 6000 analyzer (module E601) [32].

### Identifying high-risk patients using machine learning

To automatically identify the patients with a high risk of overt CVD, we investigated biochemical (8 parameters), demographical [2], clinical [17], carotid ultrasound [10], arterial stiffness-related [2] and steatosis stage in elastography [3] parameters (42 parameters in total)—the parameters are summarized in Table 1. To handle the missing data, we imputed the mean values for each parameter—the percentage of patients for whom the parameter was missing never exceeded 5% of all patients (mean: 0.68%). Afterwards, the parameters underwent univariate feature ranking. First, we examined whether each feature (predictor variable) is independent of a response variable (low- vs. high-risk patient) by using individual chi-square tests. Then, the parameters were ranked using the *p*-values of the chi-square test statistics—here, the importance of a feature is quantified as $(-\mathrm{log}(p))$, therefore a large score indicates that the corresponding predictor is important. The subsets of the most discriminative predictors were selected, and they were utilized to fitted the multiple logistic regression classifiers. Additionally, we fitted the classifiers over the principal components (PCs) extracted using PCA followed by the parallel analysis to determine the significant PCs [33]. For each model, we investigated the relationship between the model’s ability to correctly classify low- and high-risk patients using the ROC curve analysis, and we calculated the area under the ROC curve (AUC) as the summary metric to quantify the diagnostic ability of the corresponding classifier. To obtain the optimal cut-point value from each ROC curve, we exploited the (i) Index of Union (IoU), (ii) the closest to (0, 1) criteria (referred to as the Distance technique) and (iii) the Youden index methods [34]. For the selected cut-point values, we reported not only sensitivity and specificity of the corresponding classifier, but we also calculated its positive and negative predictive value (PPV and NPV, respectively), and the percentage of correctly classified low- and high-risk patients. The clinical utility of the developed ML models was investigated in the decision curve analysis. GraphPad Prism 9.4.1 was used for principal component, parallel analysis and other statistical processing, whereas MATLAB R2021b was exploited for feature selection (the fscchi2 function).

## Results

**Fig. 1:**
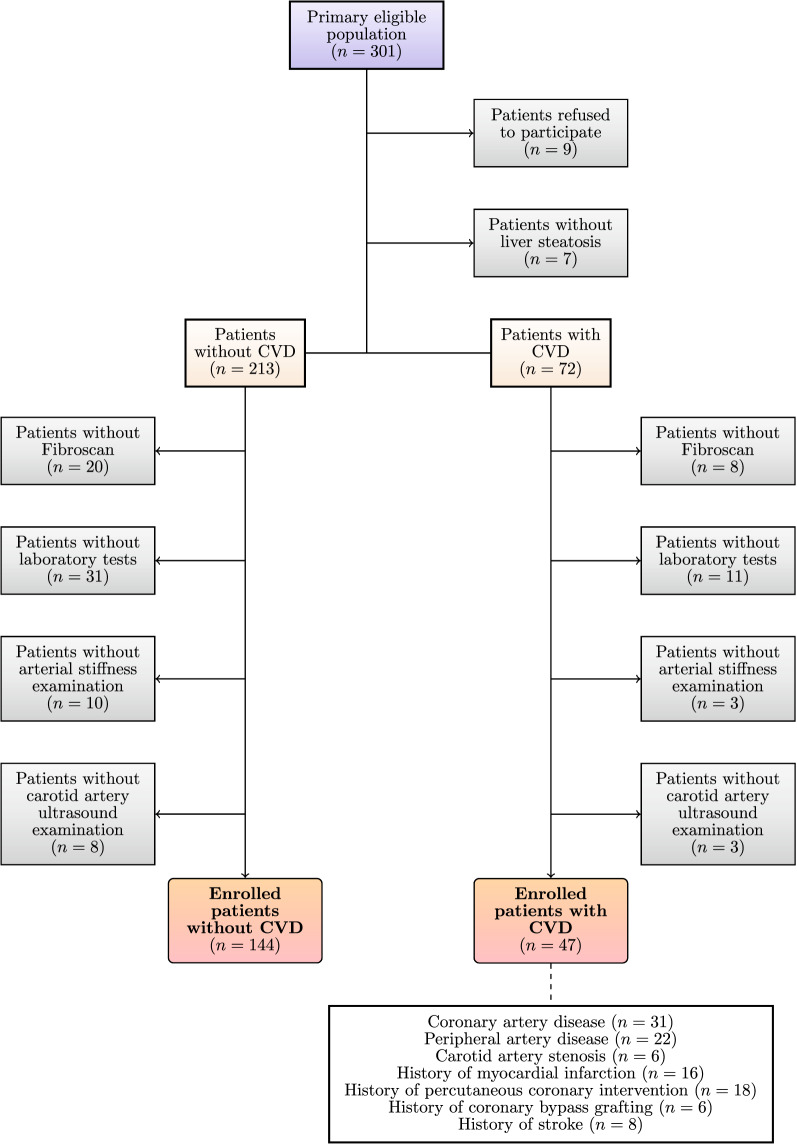
Patient flow. Out of 301 potentially eligible patients, 191 patients fulfilled the inclusion criteria (144 without CVD and 47 with CVD). CVD – cardiovascular disease

There were 301 potentially eligible patients identified, and from these, only 191 individuals (mean age 58, SD 12, median: 60, IQR: 15 years; 46% female) fulfilled the inclusion/exclusion criteria for the study (Fig. 1). The patient characteristics are gathered in Table 1.

In feature selection, we focused on Top-5, Top-10, and Top-15 most important predictors (with the 5, 10, and 15 highest importance scores, respectively) which are summarized in Table 2. The most important 5 clinical variables included hypercholesterolemia, the plaque score of the left internal carotid artery, plaque score of the right internal artery, duration of T2DM and plaque area of the right internal carotid artery, which were all positively associated with overt CVD.

**Table 2:** The 15 most discriminative features (with the largest importance scores)

Parameter	Importance score	Spearman’s (r)	p-value
Hypercholesterolemia	15.96	0.38	< 0.0001
Plaque score L	12.68	0.41	< 0.0001
Plaque score R	10.94	0.38	< 0.0001
Duration of T2DM	10.65	0.32	< 0.0001
Plaque area R	10.00	0.34	< 0.0001
Age	8.60	0.37	< 0.0001
Plaque area L	8.36	0.35	< 0.0001
Betabloker	7.66	0.25	0.0004
T2DM	7.08	0.24	0.0008
Hypertension	6.83	0.24	0.0010
cfPWV	6.64	0.28	< 0.0001
ALT	4.69	–0.15	0.0329
eGFR	3.51	–0.28	< 0.0001
HbA1c	3.19	0.22	0.0025
Obesity	3.17	–0.15	0.0421

We report the two-tailed p-values for nonparametric Spearman correlation to verify if the correlation is due to random sampling (if p < 0.05 this hypothesis can be rejected)

*T2DM* type 2 diabetes mellitus, *cfPWV* carotid-femoral pulse wave velocity, *ALT* alanine aminotransferase, *eGFR* estimated glomerular filtration rate, *HbA1c* hemoglobin A1c

**Fig. 2:**
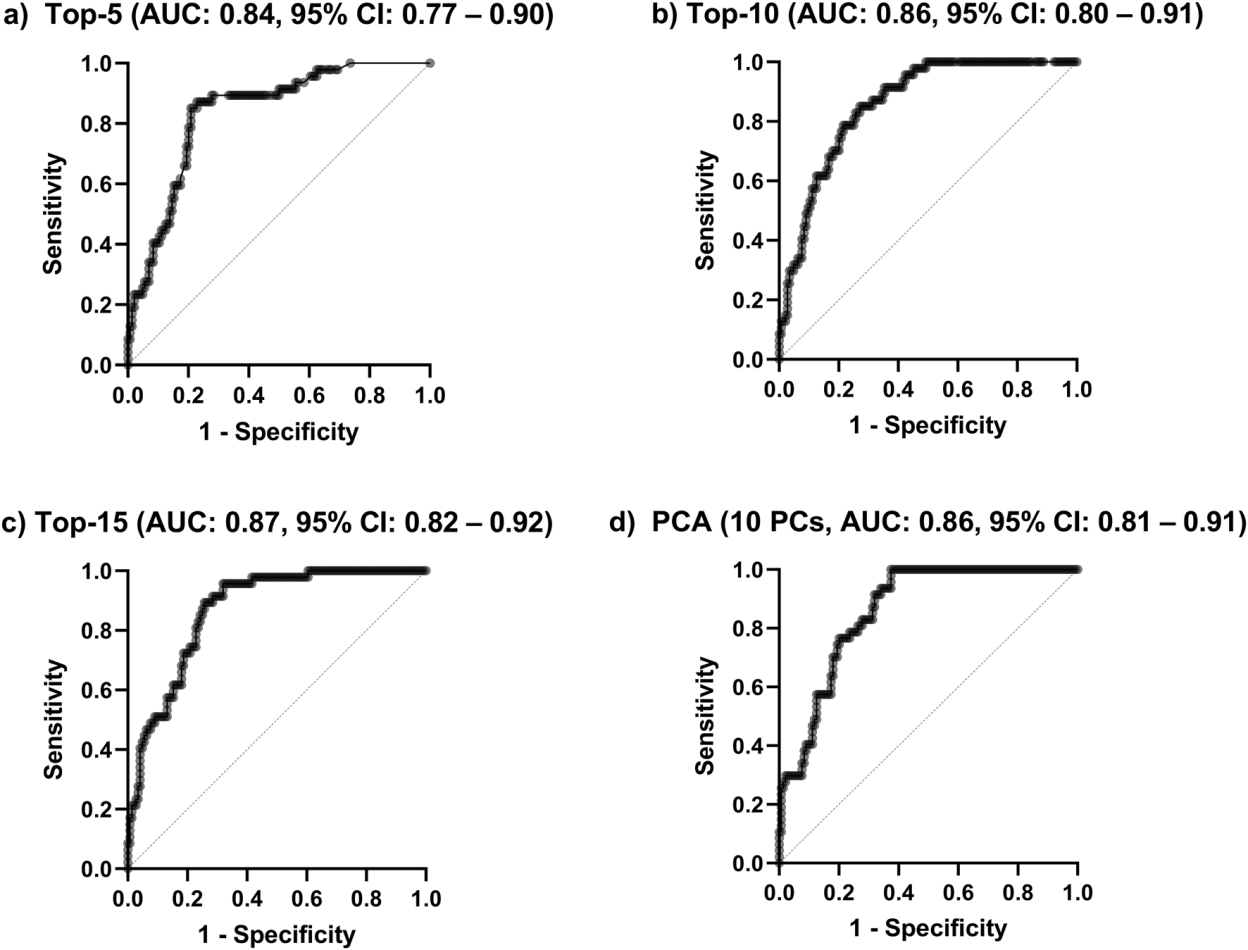
The ROC curves obtained using the multiple logistic regression classifiers over the **a** Top-5, **b** Top-10, and **c** Top-15 most discriminative patient parameters (according to their important scores), and over **d** 10 principal components (PCs). We report the area under the ROC curve (AUC) for each classifier

We investigated the performance of the multiple regression classifier fitted to ten PCs extracted by PCA, as ten PCs were found significant in the parallel analysis. The ROC analysis (Fig. 2) of the models fitted over the selected parameters, alongside the extracted PCs revealed that the highest AUC amounted to 0.87 (95% confidence interval [CI]: 0.82–0.92, *p* < 0.0001) for the model fitted to 15 most discriminative features (Top-15), whereas the worst AUC was 0.84 (95% CI 0.77–0.90, *p* < 0.0001) for the classifier operating on 5 features with the highest importance scores (Top-5). The multiple regression model fitted over 10 PCs resulted in AUC of 0.86 (95% CI 0.81–0.91, *p* < 0.0001)–utilizing 10 most important predictors led to the same AUC of 0.86 (95% CI 0.80–0.91, *p* < 0.0001). Once the optimal cut-point values have been elaborated for all models, the results were further analyzed—the performance (specificity, sensitivity, percentage of all correctly classified [CC] patients, percentage of CC low- and high-risk patients reported separately, positive and negative predictive values) of the multiple regression classifiers for the cut-point values determined using the i) IoU, ii) Distance and iii) Youden methods are reported in Table 3. The best machine learning model, as quantified by CC All and operating over five most discriminative features correctly identified 114/144 (79.17%) low-risk patients and 40/47 (85.11%) high-risk patients (Top-5 with the cut-point selected using the IoU and Distance methods).

**Table 3:** The performance of the multiple regression classifiers

Cut-point	Top-5		Top-10		Top-15			PCA (10 PCs)	
	i, ii	iii	i, iii	ii	i	ii	iii	i, ii	iii
Specificity	0.79	0.77	0.73	0.78	0.75	0.74	0.68	**0.80**	0.63
Sensitivity	0.85	0.87	0.85	0.79	0.87	0.89	0.96	0.77	**1.00**
CC low-risk [%]	79.17	77.08	72.92	78.47	75.00	74.31	68.06	**79.86**	62.50
CC high-risk [%]	85.11	87.23	85.11	78.72	87.23	89.36	95.74	76.60	**100.0**
CC All [%]	**80.63**	79.58	75.92	78.53	78.01	78.01	74.87	79.06	71.73
PPV [%]	**57.14**	55.41	50.63	54.41	53.25	53.16	49.45	55.38	46.53
NPV [%]	94.21	94.87	93.75	91.87	94.74	95.54	98.00	91.27	**100.0**

The best results are boldfaced, whereas the second best are underlined

*CC* correctly classified, *PPV* positive predictive value, *NPV* negative predictive value

**Fig. 3:**
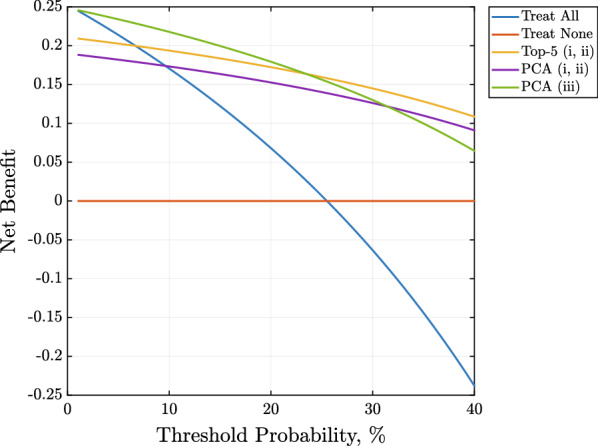
Decision curve analysis showing clinical utility of using the three best-performing ML models according to all classification performance metrics. One top-performing logistic regression classifier operated on top-5 most discriminative features with the optimal cut-point value selected using the i) Index of Union (IoU) and ii) the closest to (0, 1) criteria (yellow line), whereas two operated on 10 principal components with the optimal cut-point value selected using i) Index of Union (IoU) and ii) the closest to (0, 1) criteria (violet line) method, and using iii) the Youden index method (green line)

In Fig. 3, the clinical utility of the three best-performing ML models (according to all quality metrics gathered in Table 3) was investigated. In general, all classifiers had significantly better clinical utility (above the probability threshold of 10%) in terms of net benefit than the two alternative treatment strategies, ie. treat all or none. The logistic regression model operating on top 5 most discriminative features outperformed two other ones (exploiting 10 principal components) above the probability threshold of 25%.

## Discussion

In this study our principal findings are as follows: (*i*) we determined the most discriminative patient's parameters which can be used to build a ML model for identifying MAFLD patients with high CVD risk, (*ii*) we showed that using five interpretable and easy-to-obtain clinical parameters (including hypercholesterolemia, the plaque score of the left internal carotid artery, plaque score of the right internal artery, duration of T2DM and plaque area of the right internal carotid artery) is enough to elaborate well-generalizing logistic regression classifiers to the above-mentioned task; and (*iii*) we demonstrated better clinical utility of the developed ML models when compared to the “treat all” and “no treatment” strategies.

Shortly after the new definition of the fatty liver has emerged, it is still unknown how it will influence the clinical practice. Indeed, this does not represent a simple change in the nomenclature: differently from NAFLD, MAFLD can be recognized in patients who present with fatty liver and dysmetabolism, even when alcohol intake is reported yet not in lean ones without metabolic comorbidities [35]. It has been shown that NAFLD contributes to subclinical atherosclerosis [36–38] and that there is an independent association of NAFLD and higher prevalence of CVD in patients with DM [39]. However, it is still unclear if it is a direct effect of NAFLD per se or is it just due to the cardiometabolic risk factors shared between NAFLD and CVD [4]. It has been proved recently that presence of metabolic dysfunction, rather than alcohol consumption, may be the element which could be responsible for the superiority of MAFLD over NAFLD for discriminating worsening of atherosclerotic CVD risk in patients with fatty liver [13]. That is why it seems justified to look closer at risk factors of CVD in patients with MALFD.

In this study, 25% of relatively young patients with the Fibroscan-confirmed MAFLD presented with CVD. We have carefully reviewed the patients’ medical documentation and, surprisingly, none of the patients presented with a history of heart failure which accounts for about 2% of world adult population [40, 41]. It is, however, possible since – as the epidemiological data suggest – there is still a high number of unrecognized heart insufficiency cases [42, 43].

The feature analysis indicated that simple to obtain in everyday clinical practice parameters, such as carotid ultrasound, and clinical and biochemical ones can be discriminative in patients with overt CVD. This is very important from the practical point of view because before overt CVD occurs there is a long period of the silent disease presence and knowing the patient's parameters which could be potentially associated with overt CVD gives the clinicians a chance to improve the prevention and screening methods of CVD.

The ML models operating on such features achieved high predictive performance with the AUC values ranging from 0.84 to 0.87 (Fig. 2). Similarly, in a recent study, Oh et al. [44] analyzed the Korean national epidemiological data and demonstrated that the proposed classifiers can achieve a comparable performance (with AUC exceeding 0.85). Oh et al. determined that the most significant risk factors of CVD were age, gender, and hypertension, and they identified the positive correlation with hypertension, age, and BMI, and the negative correlation with the gender, alcohol consumption and monthly income [44]. Another study by Alaa et al. revealed that there are easy to collect, non-laboratory predictors of CVD, such as self-reported health ratings and usual walking pace which could be used in practice [45].

In our study, the top 5 most discriminative features (hypercholesterolemia, the plaque score of the left internal carotid artery, plaque score of the right internal artery, duration of diabetes and plaque area of the right internal carotid artery; are positively associated with overt CVD) could correctly identify 85.11% patients who present with CVD. While the highest score was seen for traditional risk factors, such as hypercholesterolemia and the duration of diabetes, there were also plaque related risk factors (both the plaque score and the plaque area) when taking into account top 5 most discriminative CVD features. The plaque score has been indeed identified as a factor associated with the long-term coronary artery disease risk in middle-aged asymptomatic individuals [46, 47]. Hypercholesterolemia, contrary to the duration time of diabetes, is a modifiable traditional risk factor which stress the necessity to treat this metabolic abnormalities underlined in both cardiology as well as diabetology guidelines for the management of patients with diabetes [48, 49].

When looking closer at the top 10 features which could discriminate the vulnerable patients, these are again the traditional risk factors—like being diagnosed with T2DM and hypertension—but also the use of betablocker, with all the features being positively associated with CVD. An interesting parameter which was among the top 15 ones associated with CVD is the parameter related to the arterial stiffness which is already recognized as the one which improves cardiovascular event prediction [50], and this one was also positively associated with CVD. On the other hand, other top 15 parameters (including ALT, eGFR and obesity) were negatively associated with overt CVD. Since it is understandable in relation to the kidney function expressed as eGFR which is a known risk factor of CVD, the negative association of ALT and obesity is surprising. The explanation of the negative association of obesity with CVD may be that nowadays there is a high emphasis on a healthy lifestyle, and patients who participated in our study were interested in their health since they answered the advertisement. Thus, it is also possible that they have already lost weight just before the participation in the study.

The results obtained for PCA indicated that the best-generalizing multiple regression classifiers were elaborated using interpretable features, and the models operating on the five most important features outperformed all other classifiers (exploiting more features and PCs) for the selected cut-point values, and correctly classified 80.63% of all patients (Table 3). This observation was further manifested while analyzing the clinical utility of the three best ML models – for the probability threshold of 25%, a logistic regression classifier operating on 5 patient parameters outperformed two other models, both exploiting 10 PCs which are more challenging to interpret in clinical practice. Such models directly operating on patient parameters and uncovering their interrelationships with the CVD risk are not only easier to interpret, but also are they less likely to overfit to our relatively small patient cohort. Finally, the results show that the ROC curve analysis may effectively lead to the classifiers with higher specificity or sensitivity, depending on the clinical scenario.

Most of the studies targeting the prediction of the CVD risk focus on the self-reported patient parameters which are not necessarily proved by medical documentation, hence may lead to biased outcomes. In our study, we have addressed this issue, and we analyzed a very well-defined population of patients whose concomitant diseases were medically documented. We used ML, which has been increasingly used to assess the risk of adverse outcomes in chronic disease states, outperforming simple clinical risk assessments [46, 47]. Most importantly, we are unaware of any studies to date which has assessed the risk factors in patients with MAFLD, let alone with the use of novel approaches such as ML.

### Limitations

We are aware that there are important limitations of our study, which was a proof of concept for a wider program of work. This would include larger cohorts and further external validation, as well as prospective observations for all of them. Also, the patients were not asked whether they had lost their weight before participating in the study, which could explain the negative association of obesity with CVD. An important aspect of cardiovascular pharmacologic management is antiplatelet treatment. However, we were unable to gather reliable information related to this type of treatment in patients without CVD, hence we excluded this parameter from the analysis due to a large amount of missing data. Moreover we included patients with at least 50% of carotid artery stenosis qualifying them as those with overt CVD since 50% is a cut point of stroke related to large artery atherosclerosis [51–53]. In this context, one should notice that the plaque score has been identified as one of the top 5 risk factors related to CVD so this information potentially could be biased. Finally, exploiting the recent deep learning advances which established the state of the art in various medical data analysis tasks could help us further improve the classification performance of the CVD patients, but it would also require focusing on the interpretability of such large-capacity learners, to make their deployment in clinical setting much easier [48, 49].

## Conclusion

The use of a ML approach demonstrated high performance in identifying MAFLD patients with high CVD risk based on the subset of the most discriminative, interpretable and easy-to-obtain patient's parameters. Our approach has the potential to facilitate timely diagnoses and management of prevalent CVD risk in patients who present with MAFLD in routine clinical practice.

## Data Availability

The datasets used and/or analyzed during the current study are available from the corresponding author on reasonable request.

## Abbreviations

ACC: American College of Cardiology
ACEi: Angiotensin converting enzyme inhibitor
AHA: American Heart Association
ALT: Alanine aminotransferase
ARB: Angiotensin receptor blocker
AST: Aspartate aminotransferase
AUC: Area under the receiver operating characteristic curve
BMI: Body mass index
BP: blood pressure
CABG: Coronary bypass grafting
CAP: Controlled attenuation parameter
CC: Correctly classified
CCA: Common carotid artery
CDD: Color Doppler Duplex
cfPWV: Carotid-femoral pulse wave velocity
CI: Confidence interval
CKD-EPI: Chronic Kidney Disease Epidemiology Collaboration
crPWV: Carotid-radial pulse wave velocity
CVD: Cardiovascular disease
ECA: External carotid artery
eGFR: Estimated glomerular filtration rate
HbA1c: Hemoglobin A1c
HDL-C: High-density lipoprotein cholesterol
HOMA-IR: Homeostatic Model Assessment of Insulin Resistance
HPLC: High-performance liquid chromatography
ICA: Internal carotid artery
IoU: Index of Union
IQR: Interquartile range
IMT: Intima-media thickness
LSM: Liver stiffness measurement
MAFLD: Metabolic - associated fatty liver disease
ML: Machine learning
NAFLD: Non-alcoholic fatty liver disease
NPV: Negative predictive value
PCA: Principal component analysis
PCs: Principal components
PLT: Platelet count
PPV: Positive predictive value
ROC: Receiver operating characteristic
T2DM: Type 2 diabetes mellitus
TG: Triglycerides
VLDL-C: Very low-density lipoprotein cholesterol
WHR: Waist-to-hip ratio
